# Effect of Shrinkage Reducing Admixture on Drying Shrinkage of Concrete with Different w/c Ratios

**DOI:** 10.3390/ma13245721

**Published:** 2020-12-15

**Authors:** Mahdi Kioumarsi, Fazel Azarhomayun, Mohammad Haji, Mohammad Shekarchi

**Affiliations:** 1 Department of Civil Engineering and Energy Technology, OsloMet—Oslo Metropolitan University, 0166 Oslo, Norway; 2 School of Civil Engineering, College of Engineering, University of Tehran, Tehran 1417935840, Iran; Fazel.azarhomayun@ut.ac.ir (F.A.); shekarch@ut.ac.ir (M.S.); 3 Faculty of Civil Engineering, Semnan University, Semnan 3513119111, Iran; Mohammadhaji@semnan.ac.ir

**Keywords:** concrete, mechanical properties, shrinkage reducing admixture, water-cement ratios, artificial neural network

## Abstract

The reduction of the moisture content of concrete during the drying process reduces the concrete’s volume and causes it to shrink. In general, concrete shrinkage is a phenomenon that causes concrete volume to dwindle and can lead to durability problems. There are different types of this phenomenon, among them chemical shrinkage, autogenous shrinkage, drying shrinkage including free shrinkage and restrained shrinkage, and thermal contraction. Shrinkage-reducing admixtures are commercially available in different forms. The present study investigates the effect of liquid propylene glycol ether on mechanical properties and free shrinkage induced by drying at different water-cement (w/c) ratios. Furthermore, the effect of shrinkage-reducing admixtures on the properties of hardened concrete such as compressive and tensile strength, electrical resistivity, modulus of elasticity, free drying shrinkage, water absorption, and depth of water penetration was investigated. The results indicated that shrinkage reducing agents performed better in a low w/c ratio and resulted in up to 50% shrinkage reduction, which was due to the surface reduction of capillary pores. The prediction of free shrinkage due to drying was also performed using an artificial neural network.

## 1. Introduction

Concrete is the most widely used building material in the world, such that in some countries, the use of reinforced concrete is more common than steel structures [1,2,3]. The scope of concrete consumption encompasses everything from small concrete blocks to the spillways of dams and bridge decks. One of the biggest concerns about concrete is cracking and the factors contributing to this. One of the factors that cause cracking in concrete is drying shrinkage. The evaporation rate is high for curing concrete in environments with insufficient moisture, when water temperature is high, and in windy conditions. Drying shrinkage is also more likely to occur in structures with a high surface-to-volume ratio. It has long been the concern of researchers in the field of concrete technology to find ways to reduce this type of shrinkage [4]. In this regard, one of the most effective methods is to use shrinkage reducing admixtures (SRA). The use of SRA increases the time it takes to reach the maximum hydration temperature in concrete and mortar, and by increasing the amount of SRA, the time it takes to reach maximum temperature increases; the application of SRA in mixtures with superplasticizers will further delay hydration reactions. Mora et al. [4] showed that SRA, even in high strength concrete (HSC), not only reduces shrinkage and cracking due to a reduction in evaporation rate, but also results in delayed maximum capillary pressure because of crescent cavity growth. The rate of evaporation and pore pressure in concrete containing SRA is lower than that of conventional concrete, and this difference results in reduced concrete discharge. Adding 1% SRA by cement weight, will reduce short-term and long-term shrinkage, and will be more effective when the internal moisture content and porosity of concrete are higher. Increasing the dosage of SRA in self-compacting concrete does not decrease workability [5]. In addition to reducing the pore solution, SRA with a highly retained moisture content leads to better internal curing [6]. The application of SRA has been found to increase the retention time of concrete and extend the initial and final setting time of concrete compared to a control specimen [7,8,9].

In addition to shrinkage reducing agents, the use of poly vinyl alcohol (PVA) fiber and volcanic ash are also effective in reducing shrinkage. These additives can increase the compressive strength of concrete, while reducing the surface stresses and strains associated with shrinkage. It should be noted that SRA reduces the tensile strength of concrete, its versatility and its compressive strength, which can be compensated for by using fiber [10,11]. Typically, SRA delays the hydration reaction at the start of concrete setting due to the presence of organic molecules. SRA molecules decrease the polarity of concrete mortar and increase specific surfaces, which result in an increase in the amount of water needed for hydration. This in turn leads to an improvement of hydration at higher ages [10,11]. SRA generally reduces the large pores of the cement matrix, delays crack initiation time, significantly reduces crack width, and is more effective than geopolymer materials [11,12].

Today, the use of soft computing in civil engineering to predict experimental results has been extended due to its high accuracy. Many researchers in the field of concrete technology have used soft computing and artificial neural networks (ANN) in particular to predict concrete properties based on experimental results. Mechanical properties of different types of concrete such as compressive strength, tensile strength, elastic modulus, and flexural strength can be predicted using soft computing methods.

The most important studies using artificial neural networks predict compressive strength in different concretes such as self-compacting concrete [13,14,15,16,17]; high-performance concrete [13,18]; recycled aggregate concrete [19,20,21,22]; cement mortars [23]; cement mortars containing nano and micro silica [24]; concrete containing rice husk ash as a partial replacement for cement and reclaimed asphalt pavement as a replacement for aggregates [25]; concrete under different temperatures [15,26,27] and relative humidity [15]; heavy weight concrete [28]; laterized concrete [29]; polymer concrete with various percentages of fly ash [30]; silica fume concrete [31]; high-strength concrete [32]; rubberized concrete [33]; clinker mortars [34]; lightweight concrete [27]; and self-consolidating high-strength concrete containing palm oil fuel ash [35].

In a study conducted by Bui et al. [18], the tensile strength of high-performance concrete (HPC) was predicted using a combination of ANN and firefly algorithm. The firefly algorithm was used to optimize biases and weights of ANN. The input parameters were curing age and cubic compressive strength, while the compressive strength of HPC was the output. The accuracy of the model was compared to other models and showed faster and better prediction [18]. The flexural strength of cement mortars containing nano and micro silica was predicted using ANN and genetic expression programming (GEP) [24]. The prediction of elastic modulus of concrete [36] and recycled aggregate concrete [37,38] was conducted by elephant herding optimization and ANN, respectively. An estimation of the compressive strength of concrete obtained by mechanical wave velocities was conducted using the ANN method [39,40]. The results of the ASTM C1012-95 testing method on sulfate attack of concretes, which were made with different cement types and pozzolanic additives, were predicted by ANN [40]. ANN was also applied to investigate the impermeability of concrete made with lightweight aggregate [41]. Hybrid artificial intelligence was used to predict foamed cellular lightweight concrete compressive strength using 418 experimental datasets. An equation was proposed based on a water cycle algorithm and its predictions were compared to other methods such as support vector regression, multiple linear regression, and artificial neural network [42]. An ANN model was also presented to estimate the autogenous shrinkage of concrete. The model was developed on the basis of 77 datasets, including specimens of traditional concrete as well as modern concrete [43].

Given that the effect of SRA on concrete shrinkage has not been extensively investigated in low, medium, and high water-cement (w/c) ratios, this study was conducted to investigate the effect of different w/c ratios on concrete containing SRA. For this purpose, six mix designs were considered and the effect of SRA on parameters such as free shrinkage, tensile compressive strength, Young’s modulus, electrical resistance, water absorption, and depth of penetration were investigated. Furthermore, an artificial neural network was applied to predict dry shrinkage of concrete based on the experimental results conducted in the study.

## 2. Experimental Program

### 2.1. Materials

The cement used in this study was Portland Cement Type II—in accordance with the ASTM C150 [44]—with a specific gravity of 3.15 N/m^3^. The chemical composition of the constitutive elements of the cement used in the study is presented in Table 1 below. Propylene glycol ether is a colorless liquid whose etheric property means that it is counted as an SRA material. Table 2 shows the properties of fine and coarse aggregates in accordance with the ASTM C33 [45], which were used to make the concrete. The superplasticizer used in this study was based on polycarboxylate ether and has a specific gravity of 1.1 N/m^3^. The size distribution of coarse and fine aggregate sand was in accordance with the standards ASTM C136 and ASTM C33 [45].

### 2.2. Specimen Preparation

In order to achieve the objectives of this study and obtain the mechanical properties of the intended mix designs, the concrete was manufactured with the desired mixing design. There are various tests to determine the properties of fresh and hardened concrete, some of which have been used in this study. For fresh concrete slump and air content and for hardened concrete compressive and tensile strengths, electrical resistance, modulus of elasticity, restrained drying, and water absorption, were obtained on the basis of different standards. Table 3 displays the types of experimental tests, geometry, and dimensions of the specimens based on the related standards.

As mentioned, the aim of this study is to investigate the effect of SRA on the drying-induced shrinkage of concrete in different w/c ratios. For this purpose, specimens in three low, medium, and high w/c ratios as well as control specimens were made. The concrete manufacturing process was as follows: Pouring sand into the mixer, adding gravel to the sand, adding cement to the aggregate, adding water to the mixture, adding SRA together with mix water which was kept from the previous steps, and, finally, adding superplasticizer with mix design water to the mixture. The concrete poured into the mold was kept for 24 h and then immersed in lime-saturated water at 23 ± 2 °C.

### 2.3. Mixture Proportion

The impact of SRA on the properties of fresh and hardened concrete was investigated in six mix designs classified in three groups. Table 4 illustrates the concrete’s composition in various mix designs. The dosage of superplasticizer was designed to achieve a slump of 15 ± 5 cm for mix designs with w/c ratios of 0.4 and 0.5. Since the slumps of the specimens with w/c ratio of 0.6 were in acceptable range, these specimens were designed without superplasticizer.

## 3. Results and Discussion

After fabricating the concrete with the intended mix design, the tests for fresh and hardened concrete were carried out. The results of the specimens and comparisons between them are presented in this section.

### 3.1. Fresh Concrete

In this study, two slump and air content tests were conducted based on the ASTM C143 and ASTM C231 standards in order to determine the effect of different percentages of SRA on the workability and air content of the manufactured concretes.

As shown in Table 5 below, the air content is about 2%–3% for fresh concrete when shrinkage-reducing admixtures are added to the concrete paste. Based on the results of previous studies, the impact of different types of commercial SRA materials on concrete workability is slightly different, but, overall, the impact of the SRA material on fresh concrete workability is negligible [46,47]. Table 5 shows that the SRA material increased the percentage of air and decreased the workability of fresh concrete. The same results are reported by Hamedanimojarrad [46].

### 3.2. Hardened Concrete

#### 3.2.1. Compressive Strength

**Figure 1:**
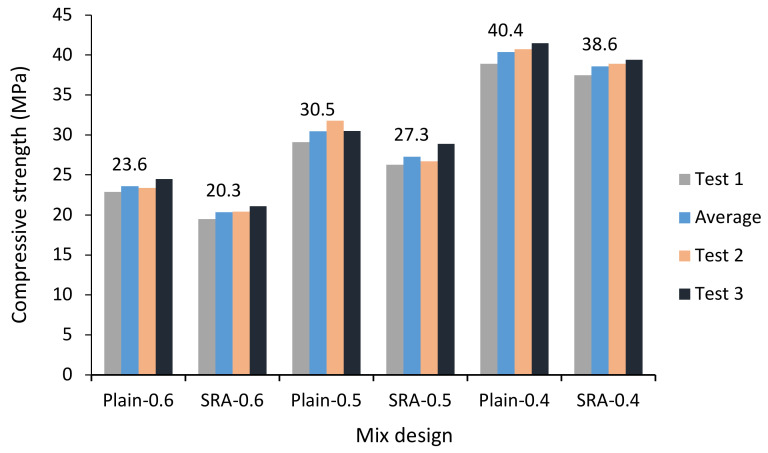
Compressive strength in specimens with three different water-cement (w/c) ratios with and without shrinkage reducing admixtures (SRA) after 28 days.

A compressive strength test was performed on cubic specimens of 150 by 150 mm in accordance with EN 12390-3 [47] at the age of 28 days. Figure 1 shows the effect of SRA on compressive strength. The average of the three compressive strengths was recorded for each mix design. SRA reduces the rate of cement hydration reactions, which consequently influences compressive strength. The mix design containing SRA reduced the compressive strength of concrete and this reduction was less with the reduction of the w/c ratio [48]. As shown in Figure 1, the SRA material in higher w/c ratios further reduces the compressive strength, which in the w/c ratio of 0.6, caused around a 14% decrease in compressive strength.

#### 3.2.2. Tensile Strength

**Figure 2:**
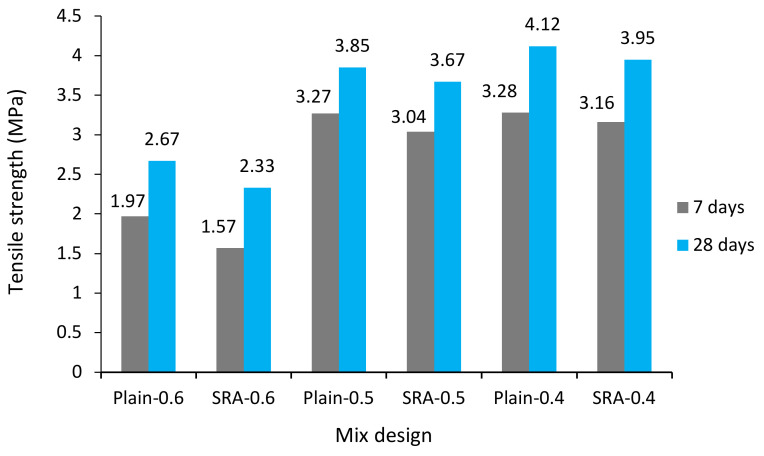
Tensile strength in specimens with three different w/c ratios with and without SRA after 7 and 28 days.

The tensile strength test was performed on cylindrical specimens measuring 300 by 150 mm in accordance with the ASTM C496 [49] standard at 28 days, and was reported as the mean tensile strength for each specimen. Figure 2 shows the effect of SRA on tensile strength. In general, the specimen containing SRA has less tensile strength, which changes with compressive strength. The average reductions in tensile strength by adding SRA to concrete were 10.33% and 7.18% for 7 and 28 days, respectively, in which the amount of reduction decreased by increasing the w/c ratio and the number of days. In other words, SRA has a greater effect on reducing tensile strength at early ages.

#### 3.2.3. Electrical Resistivity

**Figure 3:**
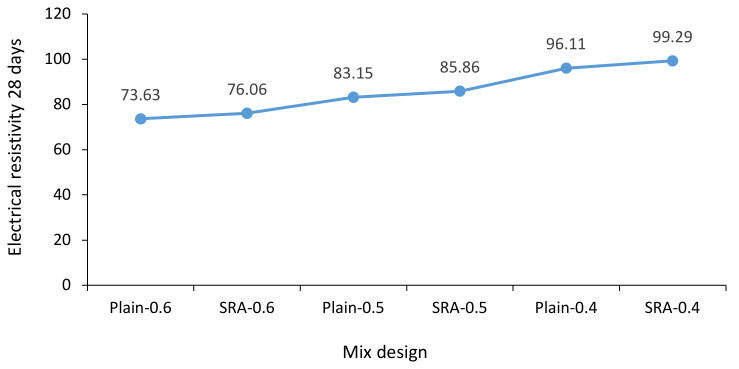
Electrical resistivity of specimens for three different w/c ratios with and without SRA after 28 days.

The electrical resistivity test was performed on 100 by 100 by 100 mm specimens according to ASSHTO T358 [48]. Figure 3 provides information on the effect of SRA on electrical resistivity. Here it can be seen that adding SRA to concrete, as well as decreasing the w/c ratio, caused an increment in electrical resistivity. However, the effect of changes to the w/c ratio was greater than the addition of SRA. The average increase in electrical resistivity from decreasing the w/c ratio was 14.26%, while this increment was 3.18% when adding SRA. Adding SRA to concrete reduces interlayer water (hydration-induced water) in the mixture, thereby reducing electrical resistivity.

#### 3.2.4. Dynamic Elastic Modulus

**Figure 4:**
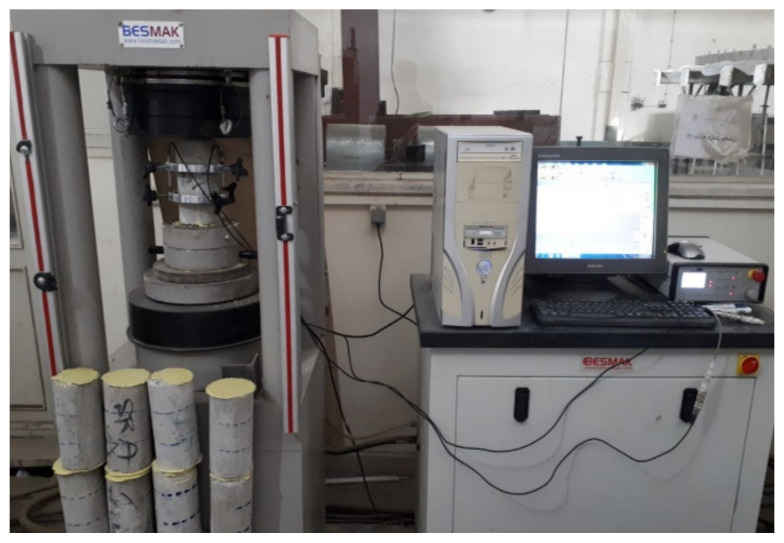
Elastic modulus measuring device.

**Figure 5:**
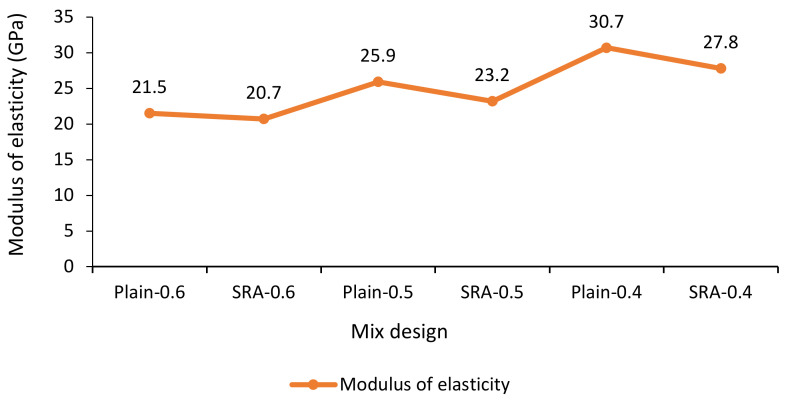
Modulus of elasticity in specimens with different w/c ratios in plain concrete and concrete containing SRA.

Young’s modulus testing was performed on specimens of 200 by 100 mm according to ASTM C469 at 28 days. Figure 4 illustrates the elastic modulus test instrument. Figure 5 shows that the w/c ratio had a greater effect on the modulus of elasticity compared to adding SRA. The highest modulus of elasticity observed in the specimens with lower w/c ratio. The specimens with SRA reduced the Young’s modulus compared to the similar specimens without SRA. These results are consistent with the results found by Haitao in 2013 [50].

#### 3.2.5. Free Drying Shrinkage

**Figure 6:**
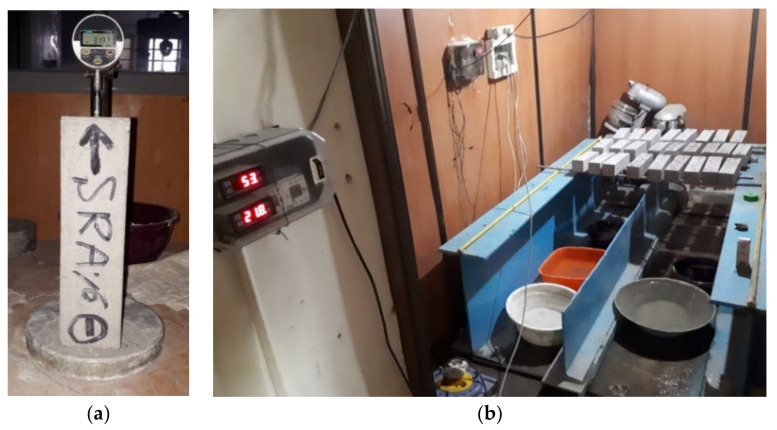
(**a**) Measurement of dry shrinkage and (**b**) specimens in the standard room.

**Figure 7:**
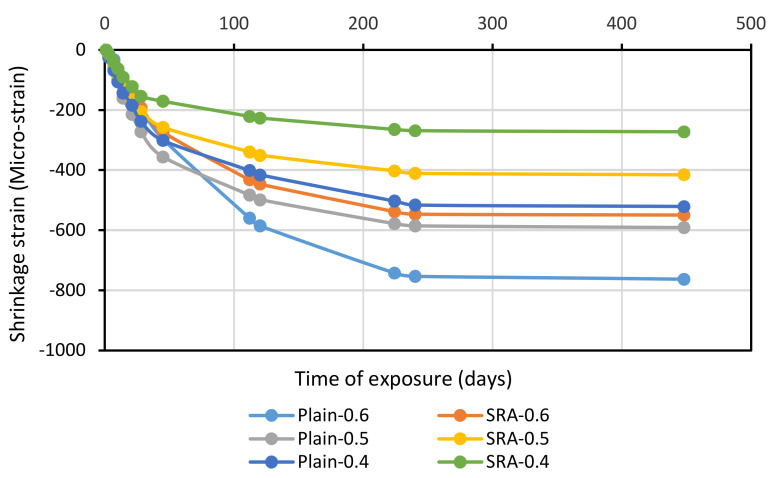
Free drying shrinkage over time for three different w/c ratios with and without SRA.

A free-drying shrinkage test was performed in line with ASTM C157, according to which the specimens were stored in a chamber with a relative humidity of 50 ± 5 and 23 ± 2. Length of the specimens was also read at intervals of 1, 4, 7, 14, and 28 days, and of 8, 16, 32, and 64 weeks [51]. Figure 6 shows the specimen in the chamber with standard conditions. The drying-induced free shrinkage test was performed on all the specimens, as shown in Figure 7. This figure shows that the free shrinkage of the mixture had a 1.5% SRA weight in a w/c ratio of 0.4, which is approximately similar to the drying-induced free shrinkage of the mixture without the SRA material in a w/c ratio of 0.5. This was due to the effect of SRA on drying-induced free shrinkage. Generally, SRA at the age of 224 days compensated for drying shrinkage by about 27%, 30%, and 50%, in the w/c ratios of 0.6, 0.5, and 0.4, respectively. Shrinkage due to a loss of excess water caused by internal stresses results in cracking. SRA reduced shrinkage stresses. It should be noted that the higher the w/c ratio, the greater the effect of the SRA material and the greater the effect on compensating for the drying shrinkage. The results indicate that reducing drying-induced shrinkage does not necessarily require a reduction in the w/c ratio and can be achieved by using SRA.

#### 3.2.6. Water Absorption

**Figure 8:**
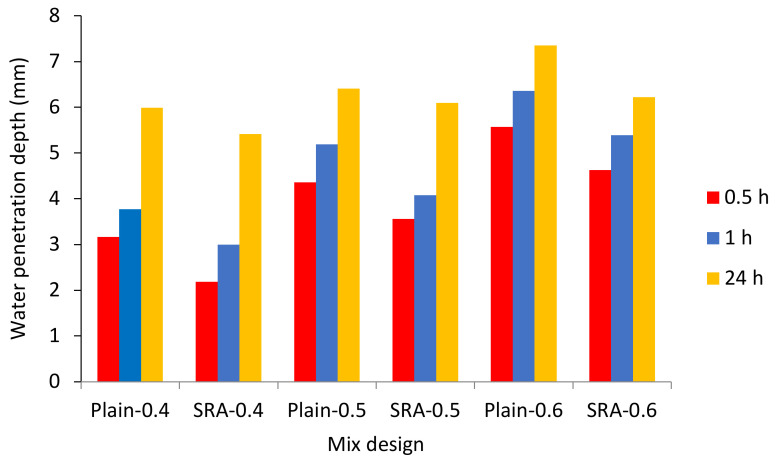
Water absorption in different mix designs after 0.5, 1, and 24 h.

**Figure 9:**
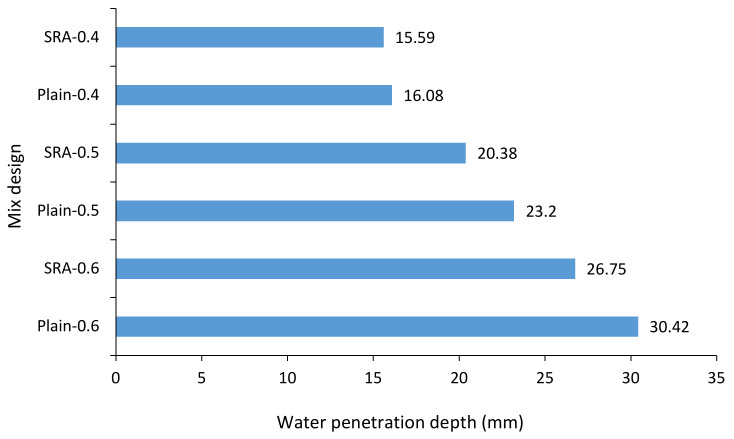
Water penetration depth in different mix designs.

The water absorption test for concrete specimens was performed according to BS 188: Part 122 [52]. Figure 8 shows the results of the comparison of changes in the mixtures’ water absorption percentage over time. As the immersion time increased, the percentage of the concrete’s water absorption increased, where the 24-h water absorption was about 2.57 times that of the half-hour water absorption. The use of SRA reduced the water absorption of the concrete. However, it is worth noting that as the immersion time increases, the impact of SRA on reducing the concrete’s water absorption increases. This may be due to the higher volume of filled pores with increased immersion time. The use of SRA reduces the surface tension stress of water with the wall of capillary pores, thereby reducing capillary suction of water into cavities, and decreasing the water absorption of concrete [53]. As the immersion time increases, more concrete pores contribute to the water absorption process. Therefore, due to the effect of SRA, the difference in water absorption percentage increases with a longer immersion time. As the w/c ratio decreases, the water absorption rate of concrete decreases. The results indicate that the effect of a w/c ratio reduction on the decrease of water absorption rate in the mixture containing SRA was approximately similar to the control design, namely the design without SRA. Furthermore, by adding SRA and increasing the w/c ratio, water penetration depth (calculated according to BS EN 12390-8 [52]) in 28 days specimens decreased, see Figure 9. The decrease in the water penetration depth is due to the effect of the SRA on the pores system of concrete.

### 3.3. Artificial Neural Networks

Many researchers have proposed models in various fields of study using soft computing methods [54,55,56,57,58,59], especially by employing artificial neural networks [28,60,61,62,63]. Artificial neural network is an artificial intelligence (AI) based method that simulates human brain to learn machines. ANN can solve new problems using past experiences like human brain. One of the most basic neural models available is multi-layer perceptron (MLP) model, which simulates the transfer function of the human brain. An artificial neural network consists of three layers: Input, output, and processing. Each layer contains a group of neurons that are generally associated with all the neurons in the other layers. After receiving the input, each neuron processes it and transmits the result to another cell. This behavior continues until a definite result is reached, which eventually leads to a decision, process, thought, or move. This is to compare the output of a network with the output that is desired and expected. The difference between the two outputs is used to change and modify the connection weights between the network units. Learning neural networks using a feedback process are called feedforward networks. Feedforward networks proceed by decreasing the difference between the actual output and the desired output until the two outputs become the same.

In this study, ANN is performed to propose a model to predict the dry shrinkage of the tested specimens. To create a network, a total of eight parameters, including the w/c ratio, type of admixture, weight of sand, gravel, and superplasticizer, were considered as input. The dry shrinkage of concrete specimens was considered to be the output parameter. The network properties used in this model are described in Table 6 below.

**Figure 10:**
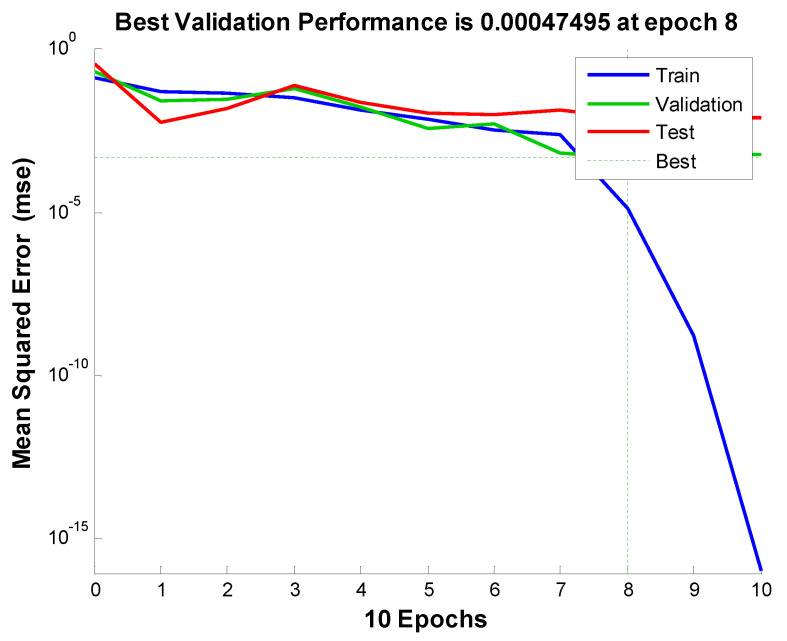
Value of mean square error (MSE) in the selected network.

**Figure 11:**
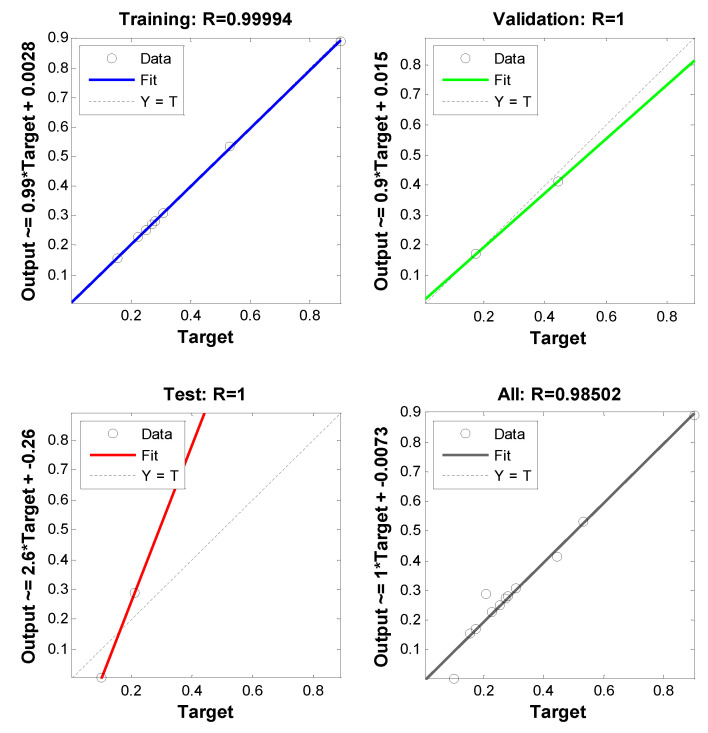
Values of regression for training, validation, testing, and the whole dataset for the selected network to predict shrinkage.

Different hidden nodes were examined (1 to 10) and their mean square error (MSE) value and the regression value for training, testing, validation, and the whole dataset, were obtained. The criterion for choosing the best network was having at least MSE (in this study the minimum MSE was equal to 0.00047), with the regression values being as close as possible to one. According to this criterion, in this study, the two-layer network was selected as the optimal network. The validation performance and training state and the values of the regressions are presented in Figure 10 and Figure 11, respectively.

The values predicted by the optimal network and its prediction errors are given in Table 7 below.

The network is selected in a way that the error values for training, validation, test, and all data are the minimum values. The other networks had shown a higher number of errors than this network. The purpose of using neural network in this study is to investigate the predicted results for shrinkage using limited available experimental data. As shown in Table 7, the maximum and minimum errors of the predicted values by artificial neural network are 0.02% and 29.77%, respectively, and the average error is 4.69%, which show the acceptable accuracy of ANN in predicting the dry shrinkage of specimens despite the low numbers of data. The highest errors have been observed in the specimen with w/c ratio of 0.4; it means that this network did not perform well in predicting the shrinkage of specimens with w/c ratio equal to 0.4. To improve the network in this case, more experimental data are required.

## 4. Conclusions

The present study investigates the effect of liquid propylene glycol ether on mechanical properties and free shrinkage induced by drying at different water-cement ratios. Furthermore, the effect of shrinkage-reducing admixtures on the properties of hardened concrete such as compressive and tensile strength, electrical resistivity, modulus of elasticity, free drying shrinkage, water absorption, and depth of water penetration was investigated. Based on the experiments, the following conclusions regarding the effect of SRA on different properties of concrete in low, medium, and high w/c ratios are drawn.

- The use of SRA reduced the slump compared to the control specimen, and the higher the w/c ratio, the greater the decrease of the slump.
- The use of SRA reduced compressive strength and tensile strength as compared to the control specimen. The higher the ratio of water to cement is, the greater the reduction in compressive strength. Using SRA caused a decrease in the Young’s modulus of the concrete and had a greater effect in a w/c ratio of 0.4.
- The use of SRA increased the electrical resistivity of the concrete, which has the same effect on the electrical resistivity of concrete in low, medium and high w/c ratios.
- Using SRA caused a decrease in the depth of penetration of water under pressure. It decreased less in a low w/c ratio compared to other ratios.
- Using SRA caused a decrease in the water absorption rate of concrete. It was observed that the effect of SRA on the reduction of water absorption of concrete is dependent on the immersion time of the concrete in water.
- The use of SRA caused a 50% decrease in free shrinkage induced by drying.
- Prediction of the dry shrinkage of specimens was performed using an artificial neural network, with low and acceptable mean error, indicating the high accuracy of ANN in predicting dry shrinkage based on experimental results.

## Figures and Tables

**Table 1:** Percentages of the cement constituents.

Composition	CaO	SiO_2_	Al_2_O_3_	Fe_2_O_3_	MgO	K_2_O	Na_2_O	C_3_S	C_2_S	C_3_A	C_4_AF
**%**	64.3	21.8	4.5	3.9	1.5	0.54	0.17	56	20	5	12

**Table 2:** Physical properties of the fine and coarse aggregate.

Properties	Coarse Aggregate	Fine Aggregate
Specific gravity (saturated surface dry) (N/m^3^)	2.56	2.55
Water absorption (%)	1.7	2.7
Physical shape	Crushed	Well-rounded

**Table 3:** Type and number of specimens prepared, and standards used.

Test	Specimen	Dimensions (mm)	Number Samples for Each Test	Standard
Air content	-	-	1	ASTM C231
Slump	-	-	1	ASTM C143
Compressive strength	Cubic	150 × 150 × 150	3	EN 12390-3
Tensile strength	Cylinder	150 × 300	2	ASTM C496
Unrestrained drying shrinkage	Prism	75 × 75 × 285	3	ASTM C157
Electrical resistance	Cubic	100 × 100 × 100	3	ASSHTO T358
Modulus of elasticity	Cylinder	100 × 200	3	ASTM C469
Water absorption	Cubic	750 × 750 × 750	3	BS 1881: Part 122

**Table 4:** Concrete components in various mix designs.

Group	Specimen Name	Cement (kg/m^3^)	Water (kg/m^3^)	w/c	Fine Aggregate (kg/m^3^)	Coarse Aggregate (kg/m^3^)	SRA % (Weight of Cement)	Superplasticizer % (Weight of Cement)
1	Plain-0.6	350	210	0.6	1151	619	-	-
	SRA-0.6	350	210	0.6	1151	619	1.5	-
2	Plain-0.5	350	175	0.5	1173	632	-	0.5
	SRA-0.5	350	175	0.5	1173	632	1.5	0.5
3	Plain-0.4	350	140	0.4	1196	644	-	0.9
	SRA-0.4	350	140	0.4	1196	644	1.5	0.9

**Table 5:** Properties of fresh concrete specimens.

Group	w/c	Specimen Name	Slump (cm)	Air Content (%)
1	0.6	Plain-0.6	21	1.6
	0.6	SRA-0.6	18	2.1
2	0.5	Plain-0.5	15	2
	0.5	SRA-0.5	12	2.4
3	0.4	Plain-0.4	15	2.6
	0.4	SRA-0.4	11	3

**Table 6:** Network properties used in this model.

Network Type	Feed-Forward Backprop
Training function	TRAINLM
Adaption learning function	LEARNGDM
Performance function	MSE
Number of layers	2
Transfer function (layer 1)	TANSIG
Transfer function (layer 2)	PURELIN

**Table 7:** Predicted shrinkages by artificial neural network along with its error.

Mix Design	w/c	SRA (%)	Fine Aggregate (kg/m^3^)	Coarse Aggregate (kg/m^3^)	Superplasticizer % (Weight of Cement)	Exp. Shrinkage	Predicted Shrinkage	Error (%)
Plain-0.6	0.6	0	1099	593	0	0.63	0.63	0.88
SRA-0.6	0.6	1.5	1099	593	0	0.30	0.3	0.23
SCA-0.6	0.6	0	1099	593	0	0.27	0.27	0.31
PRA-0.6	0.6	0	1099	593	0	0.28	0.28	0.02
Plain-0.5	0.5	0	1159	610	0.7	0.43	0.43	0.37
SRA-0.5	0.5	1.5	1159	610	0.7	0.28	0.28	0.06
SCA-0.5	0.5	0	1159	610	0.7	0.22	0.22	0.74
PRA-0.5	0.5	0	1159	610	0.7	0.25	0.25	0.347
Plain-0.4	0.4	0	1189	639	3.15	0.38	0.36	4.62
SRA-0.4	0.4	1.5	1189	639	3.15	0.24	0.29	18.61
SCA-0.4	0.4	0	1189	639	3.15	0.18	0.13	29.77
PRA-0.4	0.4	0	1189	639	3.15	0.21	0.21	0.36
